# Paired primary and metastatic lesions of patients with ipilimumab-treated melanoma: high variation in lymphocyte infiltration and HLA-ABC expression whereas tumor mutational load is similar and correlates with clinical outcome

**DOI:** 10.1136/jitc-2021-004329

**Published:** 2022-05-11

**Authors:** Mark A J Gorris, Lieke L van der Woude, Leonie I Kroeze, Kalijn Bol, Kiek Verrijp, Avital L Amir, Jelena Meek, Johannes Textor, Carl G Figdor, I Jolanda M de Vries

**Affiliations:** 1 Tumor Immunology, Radboudumc, Nijmegen, The Netherlands; 2 Oncode Institute, Nijmegen, The Netherlands; 3 Pathology, Radboudumc, Nijmegen, The Netherlands; 4 Medical Oncology, Radboudumc, Nijmegen, The Netherlands; 5 Department of Tumor Immunology, Radboudumc, Nijmegen, The Netherlands; 6 Data Science Group, Institute for Computing and Information Sciences, Radboud Universiteit, Nijmegen, The Netherlands

**Keywords:** Melanoma, Tumor Microenvironment, Immunohistochemistry, Lymphocytes, Tumor-Infiltrating, Immunotherapy

## Abstract

**Background:**

Immune checkpoint inhibitors (ICI) can lead to long-term responses in patients with metastatic melanoma. Still many patients with melanoma are intrinsically resistant or acquire secondary resistance. Previous studies have used primary or metastatic tumor tissue for biomarker assessment. Especially in melanoma, metastatic lesions are often present at different anatomical sites such as skin, lymph nodes, and visceral organs. The anatomical site may directly affect the tumor microenvironment (TME). To evaluate the impact of tumor evolution on the TME and on ICI treatment outcome, we directly compared paired primary and metastatic melanoma lesions for tumor mutational burden (TMB), HLA-ABC status, and tumor infiltrating lymphocytes (TILs) of patients that received ipilimumab.

**Methods:**

TMB was analyzed by sequencing primary and metastatic melanoma lesions using the TruSight Oncology 500 assay. Tumor tissues were subjected to multiplex immunohistochemistry to assess HLA-ABC status and for the detection of TIL subsets (B cells, cytotoxic T cells, helper T cells, and regulatory T cells), by using a machine-learning algorithm.

**Results:**

While we observed a very good agreement between TMB of matched primary and metastatic melanoma lesions (intraclass coefficient=0.921), such association was absent for HLA-ABC status, TIL density, and subsets thereof. Interestingly, analyses of different metastatic melanoma lesions within a single patient revealed that TIL density and composition agreed remarkably well, rejecting the hypothesis that the TME of different anatomical sites affects TIL infiltration. Similarly, the HLA-ABC status between different metastatic lesions within patients was also comparable. Furthermore, high TMB, of either primary or metastatic melanoma tissue, directly correlated with response to ipilimumab, whereas lymphocyte density or composition did not. Loss of HLA-ABC in the metastatic lesion correlated to a shorter progression-free survival on ipilimumab.

**Conclusions:**

We confirm the link between TMB and HLA-ABC status and the response to ipilimumab-based immunotherapy in melanoma, but no correlation was found for TIL density, neither in primary nor metastatic lesions. Our finding that TMB between paired primary and metastatic melanoma lesions is highly stable, demonstrates its independency of the time point and location of acquisition. TIL and HLA-ABC status in metastatic lesions of different anatomical sites are highly similar within an individual patient.

**Key messages**

- Thus far, no single biomarker can fully predict response to immunotherapy and consequently no biomarkers are currently used to include or exclude patients with melanoma from receiving immunotherapy.
- Tumor mutational burden (TMB) of paired primary and metastatic melanoma lesions is highly stable and correlates with clinical outcome of ipilimumab in patients with melanoma hence supporting the use of TMB of either primary or metastatic lesions as contributing factor in prediction models.
- Tumor infiltrating lymphocytes (TILs) and HLA-ABC status showed a high variation between primary and metastatic melanoma lesions, but are highly similar between metastatic lesions of different anatomical sites within an individual patient.
- HLA-ABC status in the metastatic lesion could to some extent contribute to a prediction model, but needs to be validated in independent cohorts.
- TILs differ substantially between the primary tumor and metastatic lesions, hampering its suitability for biomarker development, especially for patients with early-stage disease of whom no metastatic material is readily available.

## Background

The first Food and Drug Administration (FDA)-approved immune checkpoint inhibitor (ICI) for metastatic melanoma was ipilimumab, a monoclonal antibody that blocks the negative signaling receptor cytotoxic T-lymphocyte associated protein 4 (CTLA-4) on T cells.^1 2^ Approximately 20% of patients with metastatic melanoma respond to treatment, leading to long-term survival.^3 4^ Even higher response rates are seen with immunotherapeutic drugs blocking the programmed cell death protein 1 (PD-1) pathway that were developed subsequently,^5–7^ or combination thereof.^8^ Still, many patients with melanoma do not respond or acquire secondary resistance, leading to disease progression. Patients with intrinsically resistant disease are exposed to non-effective treatment with potential severe side effects. Studying the tumor microenvironment (TME) can help to understand the mechanisms of response and resistance and may lead to the development of strategies to improve response rates of immunotherapy.

Biomarkers are an important tool for clinical decision-making in starting targeted therapies for melanoma treatment, such as the *BRAF* mutation status.^9 10^ For ICI however, accurate prediction of response remains difficult. High tumor mutational burden (TMB)^11–14^ and programmed cell death 1 ligand 1 (PD-L1) expression^15^ are associated with response to PD-1 blocking therapy in patients with melanoma. However, objective responses are also observed in patients with melanoma with low TMB^16^ or without detectable PD-L1 expression.^15 17 18^ Thus far, no single biomarker can fully predict response to therapy^19 20^ and consequently no biomarkers are currently used to include or exclude patients with melanoma from receiving immunotherapy. Nevertheless, high TMB and microsatellite instability are FDA-approved biomarkers in other solid tumors for the treatment with PD-1 blocking therapy.^21 22^ Other factors that have been associated with response to immunotherapy in patients with melanoma are gene expression profiling,^23–26^ major histocompatibility complex (MHC) molecule expression,^27 28^ T cell receptor diversity,^29 30^ lymphocyte infiltration and other immune cell markers.^31–38^ A solution for better prediction may come from a combination of multiple biomarkers. Therefore, it is important to explore the various components that may contribute to such a composite biomarker profile.

Research focusing on biomarker discovery in the TME is highly diverse and many unanswered questions remain. When studying the TME in melanoma, mostly tumor samples from metastatic sites are studied. However, these samples originate from different anatomical sites such as skin, lymph node, and visceral organs. This organ-specific tissue in which a metastasis is located may influence the TME composition. So far, only few studies have focused on primary tumors for biomarker discovery.^39 40^ This is particularly important for patients from whom there is no metastatic tumor tissue available, as well as for the development of biomarkers for (neo)adjuvant treatment of early stage disease.^41–43^

Currently, it is unclear how comparable the TME of primary tumors and their respective metastases at distinct anatomical sites are. To predict treatment response, it is likely necessary to identify biological features that remain relatively stable over time and are also shared by both primary and metastatic lesions. In this retrospective study, we compared the TME of paired primary and metastatic lesions of patients with melanoma that were treated with ipilimumab. We studied the TMB, infiltrating lymphocyte subsets, and HLA-ABC status to determine (dis)similarities between primary and metastatic lesions derived from different anatomical sites and during the course of the disease. Tumor infiltrating lymphocytes (TILs) were investigated by multiplex immunohistochemistry (mIHC) to interrogate multiple lymphocyte subsets simultaneously and their spatial relationship to the tumor, measured by TILs within the tumor and within the invasive margin.

## Material and methods

### Patient material

Formalin-fixed paraffin-embedded (FFPE) melanoma tissue blocks (primary and metastatic melanoma lesions) of 33 patients who received first-line or second-line ipilimumab monotherapy between 2012 and 2015 at the Radboud university medical center (Radboudumc), Nijmegen or Isala hospital, Zwolle, the Netherlands were retrospectively requested at the hospital of resection/biopsy across the Netherlands. Material of one patient was not analyzed because of uncertainty about the subtype of melanoma. The other 32 patients all belong to the low cumulative solar damage group.^44^ Good quality material was obtained from paired primary and metastatic melanoma lesions in 29 patients and paired metastatic lesions in 18 patients. All metastatic lesions were obtained prior to start of ipilimumab. For studying response to ipilimumab, material of one patient was not considered, because only one dose of ipilimumab was given to continue with pembrolizumab, hence response to ipilimumab could not be determined.

### Nucleic acid extraction

Tumor surface area and percentage was estimated from H&E stained sections, disregarding surrounding stroma by a pathologist, requiring a minimum of 20% tumor cells for analysis. Based on surface area estimates, 1–10 sections of 10–20 µm thickness were cut from FFPE blocks to yield a tumor volume of ~2 mm^3^. Tumors were macrodissected, placed into Eppendorf tubes and deparaffinized using xylene. Nucleic acids, DNA and RNA, were extracted using the AllPrep DNA/RNA FFPE Kit (80234, QIAgen) following manufacturer’s instructions. Elutions were performed by washing the column two times with 20 µl and 15 µl RNAse free water for RNA and 50 µl and 30 µl buffer ATE for DNA. RNA was stored at −80°C and DNA at −20°C. DNA concentrations were measured with Qubit dsDNA HS Assay Kit (Q32854, Thermo Fisher) on the Qubit Fluorometer (Thermo Fisher). DNA integrity number (DIN) was assessed in samples containing detectable DNA concentrations on the 2200 TapeStation System (Agilent) using the genomic DNA ScreenTape (5067–5365, Agilent). A selection of samples with ranging DIN values were subjected to quality control using TruSeq FFPE DNA Library PrepQC Kit (FC-121–9999, Illumina) and KAPA SYBR FAST (KK4600, Sigma-Aldrich) on QuantStudio 3 (Thermo Fisher). Samples with a delta Cq score of preferably <6 or DIN value of ≥2.3 passed quality assessment and were proceeded with.

### TSO500 library preparation for sequencing

A selection of tumor samples was subjected to sequencing. Library preparation was performed using the hybrid capture-based TruSight Oncology 500 Library Preparation Kit (TSO500; Illumina) following the manufacturer’s protocol. DNA preparation and sequencing were performed as described before.^45^ 40–100 ng DNA was used as input for the library preparation, depending on the age and quality of the DNA sample.

### Sequencing and data analysis

Libraries were sequenced on a NextSeq 500 (Illumina), with 8–10 libraries sequenced per run (NextSeq high-output). The sequence data were processed and analyzed by the TruSight Oncology 500 Local App V.2.0 (Illumina).^45^ For TMB estimation using TSO500 software, variants are classified as somatic or germline by using bioinformatical approaches which make use of various databases (including COSMIC, gnomAD and 1000 genomes project) and also takes into account the variant allele frequencies (VAFs) of surrounding germline variants. For TMB calculation, somatic single-nucleotide variants with a VAF >5% are included. Hotspot mutations are excluded to avoid overestimation of TMB, since the gene panel is biased towards frequently mutated genomic regions (cancer-related genes). TMB values are expressed in non-synonymous mutations per mega base of DNA (nsmut/Mb). Clonal relationship between primary and metastasis was studied by analyzing a set of melanoma-associated genes using TSO500 data: BAP1 (NM_004656), BRAF (NM_004333), CTNNB1 (NM_001904), EIF1AX (NM_001412), GNA11 (NM_002067), GNAQ (NM_002072), HRAS (NM_005343), KIT (NM_000222), NF1 (NM_001042492), NRAS (NM_002524), RAC1 (NM_018890), SF3B1 (NM_012433), TERT promoter (NM_198253) and TP53 (NM_000546). Subsequently, additional genes were analyzed for clonality analysis and to study its influence on HLA-ABC expression and lymphocyte infiltration: B2M (NM_004048), APC (NM_000038), AXIN1 (NM_003502) and PTEN (NM_000314).

### mIHC

Sections of 4 µm thickness were cut from FFPE tissue blocks and mounted on SuperFrost Plus glass slides (900226, VWR). Slides were subjected to sequential staining cycles^46^ using an automated platform with Opal 7-color Automation IHC Kit (NEL801001KT; PerkinElmer) on the BOND RX IHC & ISH Research Platform (Leica Biosystems).^47 48^ All heat induced epitope retrievals on the BOND RX were performed with Bond Epitope Retrieval 2 (AR9640, Leica Biosystems) for 20 min at 95°C. Blocking was performed with antibody diluent. All primary antibodies were incubated for 1 hour at RT. All secondary antibodies were incubated for 30 min at RT. For the detection of lymphocytes, the following sequence of mIHC was performed; anti-CD45RO (Thermo Scientific, MS-112, clone UCHL-1, 1:3000) with Opal620, anti-CD8 (Dako, M7103, clone C8/144B, 1:200) with Opal690, anti-CD20 (Thermo Fisher, MS-340, clone L26, 1:600) with Opal570, anti-CD3 (Thermo Fisher, RM-9107, clone RM-9107, 1:200) with Opal520, FOXP3 (eBioscience Affymetrix, 14–4777, clone 236A/E7, 1:100) with Opal540. A 4-color mIHC panel for the detection of MHC class I (MHC-I) and indoleamine 2,3-dioxygenase 1 (IDO-1) was performed manually. Heat induced epitope retrievals were performed with EnVision FLEX target retrieval solution (pH 9, K8004, Dako). The following sequence of mIHC was performed; anti-HLA-ABC (Abcam, ab70328, clone EMR8-5, 1:1000) with Opal520 and anti-IDO-1 (Merck, MAB10009, Clone 1F8.2, 1:100) with Opal570. Manual washing steps were performed with EnVision FLEX Wash Buffer (DM831, Dako). The last staining cycle for both panels was performed with a melanoma specific antibody cocktail (melmix) consisting of anti-HMB-45 (Cell Marque, 282M-9, clone HMB-45, 1:600), anti-Mart-1 (Cell Marque, 281M-8, clone A103, 1:300), anti-Tyrosinase (Cell Marque, 344M-9, clone T311, 1:200) and anti-SOX-10 (Cell Marque, 383R-1, clone EP268, 1:500) and Opal650 to visualize tumor tissue. Slides were counterstained with DAPI and mounted with Fluoromount-G (SouthernBiotech, 0100–01).

### Imaging and analysis

**Figure 1:**
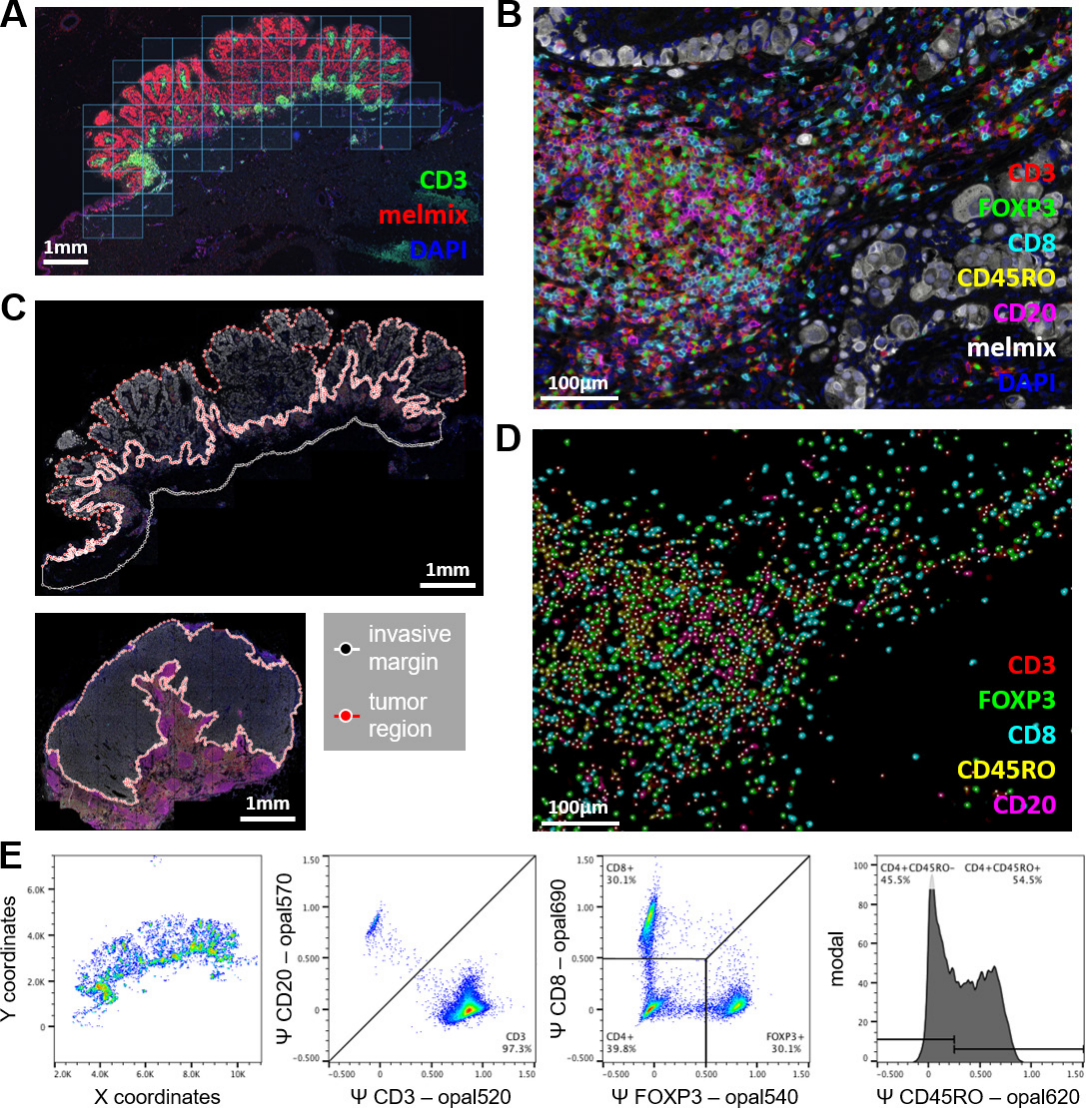
Multiplex imaging and data processing of the melanoma tumor microenvironment. (A) Multiplex stained slides were prescanned at 4× and tiles of 20× multispectral imaging were selected using Phenochart. Tissues were scanned completely with surrounding tiles for the invasive margin. (B) Images were unmixed using inForm to visualize the markers: CD3 (red), FOXP3 (green), CD8 (cyan), CD45RO (yellow), CD20 (magenta), melmix (white), DAPI (blue) and autofluorescence (not shown). (C) ROI for tumor and, if possible, invasive margin of ~0.5 mm thickness, were manually selected on scanned tissues. Upper image represents the primary tumor and the lower image a lymph node metastasis. (D) Lymphocytes were identified based on marker expression using a machine-learning algorithm. Colors show inferred intensity of surface marker expression for cells detected by the algorithm. (E) Lymphocytes that are recognized by the machine-learning algorithm were exported as FCS file and were phenotyped based on inferred marker expression. (D) using a gating strategy that first separated T-cells (CD3^+^CD20^−^) from B-cells (CD3^−^CD20^+^). Next, T cells were further separated into cytotoxic T cells (CD3^+^CD8^+^FOXP3^−^), regulatory T cells (CD3^+^CD8^+^FOXP3^-^) and helper T cells (CD3^+^CD8^−^FOXP3^−^). Cytotoxic T cells, regulatory T cells, and helper T cells can be further divided by CD45RO expression (CD45RO signal on helper T cells are shown in this example). FCS, flow cytometry standard; ROI, region of interest.

Stained tissue sections were pre-scanned at 4 times magnification using the Automated Quantitative Pathology Imaging System (Vectra V.3.0.4, PerkinElmer) for the seven-color panel or at 10 times for the four-color panel. Prescans at 4 times magnification were used to select a region to completely cover the tumor and the invasive margin for multispectral imaging using Phenochart (V.1.0.9, PerkinElmer) at 20 times magnification (figure 1A). Spectral unmixing of Opal fluorophores, DAPI and autofluorescence was performed with inForm software (V.2.2.1, PerkinElmer) (figure 1B). Distinct tissue regions and populations of lymphocyte subsets could be visually detected based on CD20, CD3, CD8, and FOXP3. Tissue regions of interest (ROI) were manually selected by dividing the tissue into a tumor region and, wherever possible, an invasive margin surrounding the tumor of ~0.5 mm thickness using an in-house developed program (figure 1C). Necrotic tumor areas were excluded. An invasive margin was not selected in metastatic lesions within lymph nodes (figure 1C, bottom image), because no distinction could be made between lymphocytes belonging to the normal lymph node tissue and tumor attracted lymphocytes. In other metastatic lesions that did not contain stromal tissue surrounding the tumor, no invasive margin was selected either. When necessary, ROI drawing was guided by H&E slides (online supplemental figure 1). Immune cells were identified using a machine learning (ML) algorithm that was developed for the recognition of immune cells in a range of different tumor types (figure 1D).^49^ ML-recognized immune cells were converted into Flow Cytometry Standard files and were further phenotyped using FlowJo software (V.10; Tree Star) into total T cells (CD3^+^) and B cells (CD20^+^), total T cells were further divided into cytotoxic T cells (CD3^+^CD8^+^), T regulatory cells (CD3^+^FOXP3^+^), T helper cells (CD3^+^CD8^−^FOXP3^−^) (figure 1E). The addition of CD45RO in the phenotyping allowed for dividing cytotoxic T cells, T regulatory cells and T helper cells into CD45RO^+^ and CD45RO^−^ categories. Phenotyped immune cell data were expressed in density by dividing the absolute numbers with the surface area of the tissue region (tumor, invasive margin, or tumor + invasive margin) or in percentages of total T lymphocytes for CD45RO status. HLA-ABC and IDO-1 expression were assessed from the 10 times magnified pre-scan in Phenochart by two blinded researchers that reached an agreement. Representative multispectral images were taken at 20 times magnification. HLA-ABC was scored on the tumor using four different categories: no loss of HLA-ABC, (partly) dim expression of HLA-ABC, <50% loss of HLA-ABC and >50% loss of HLA-ABC. No loss of HLA-ABC and (partly) dim expression of HLA-ABC was regarded as ‘no loss of HLA-ABC’ in the final analysis. In some analyses, <50% loss of HLA-ABC and >50% loss of HLA-ABC was also taken together as ‘loss of HLA-ABC’. IDO-1 was not further analyzed for this study as only few tumor samples showed clear expression of this marker on tumor cells. Stromal expression of IDO-1 was observed but not further scored or analyzed due to technical difficulties.

10.1136/jitc-2021-004329.supp3 Supplementary data

### Statistics

Progression-free survival (PFS) was calculated from the start of ipilimumab to the date of progressive disease according to the Response Evaluation Criteria in Solid Tumors V.1.1. Clinical benefit was defined as a complete response, partial response, or stable disease for at least 6 months. The data cut-off point was January 2021. Correlations were measured using linear regression on log-transformed data displaying R^2^ (GraphPad Prism V.8) or intraclass coefficient (ICC) using an absolute agreement definition and single measures calculated with SPSS (V.25, IBM). ICC is a measure of inter-rater agreement that ranges from 0 (no agreement) to 1 (perfect agreement). Agreement of TMB and TIL (subset) density between different tumor lesions were interpreted as followed:<0.4—poor, 0.4–0.59—modest, 0.6–0.75—good, and ≥0.75—very good. Data graphs were generated using GraphPad Prism V.8. CD45RO status on total CD3^+^ T cell among primary and metastatic lesions was analyzed using a mixed-effects analysis and post-hoc Tukey’s multiple comparisons test. Graphs with error bars are presented as mean with SEM. Lymphocyte densities were log-transformed and significance between loss and no loss of HLA-ABC, benefit, and no benefit groups was calculated using unpaired t-test. The significance of PFS between loss and no loss of HLA-ABC groups was calculated using Mann-Whitney test. TMB data were log-transformed and significance between benefit and no benefit groups was calculated using unpaired t-test with Welch’s correction. P values ≤0.05 were considered significant.

## Results

### TMB between primary and metastatic melanoma lesions is highly similar

The mutational burden of a tumor is a promising biomarker for the response to ICI.^22^ To determine whether melanomas change in overall TMB between the primary tumor and metastases, DNA was isolated from primary and metastatic lesions and TMB values in these lesions were compared (online supplemental table 1). We were able to isolate DNA of sufficient quality of 80% of tumors, even from tumor FFPE blocks of more than >10 years old. Mutation analysis in a selection of genes confirmed the likelihood of a clonal relationship between primary and metastatic lesions (online supplemental table 2).

10.1136/jitc-2021-004329.supp1 Supplementary data

10.1136/jitc-2021-004329.supp2 Supplementary data

**Figure 2:**
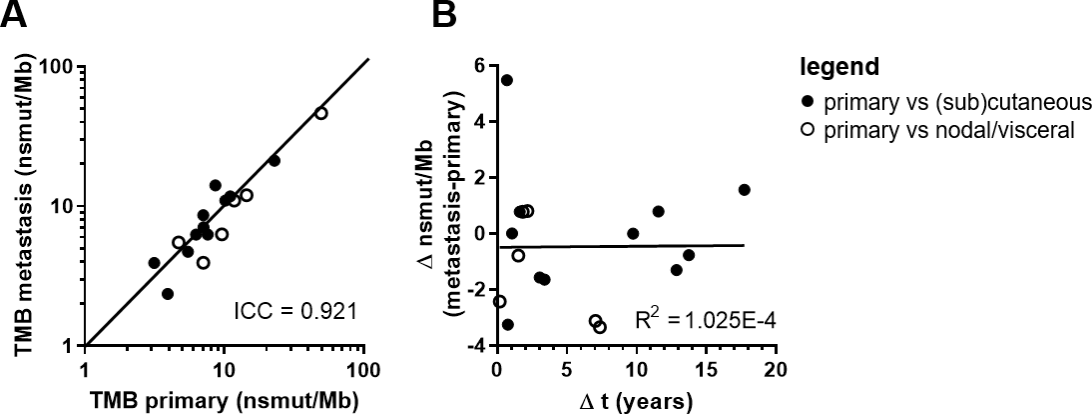
TMB value in paired primary and metastatic melanoma samples. (A) Correlation plot of primary tumor TMB versus metastatic tumor TMB. Values are displayed in nsmut/Mb. Concordance is shown and quantified using the ICC. (B) Correlation plot of the difference in TMB between paired metastatic and primary melanoma sample versus the time between these samples. Closed dots represent paired primary cutaneous tumors with metastatic tumors at (sub)cutaneous locations. Open dots represent paired primary cutaneous tumors with metastatic tumors at nodal or visceral locations. n=17. ICC, intraclass correlation coefficient; nsmut/Mb, non-synonymous mutations/mega base; TMB, tumor mutational burden.

A strong agreement was observed between the TMB of the primary and metastatic lesion of the same patient (ICC=0.921, 95% CI: 0.800 to 0.970, p=3.22E-8; figure 2A). This agreement was independent of the anatomical location of the metastasis. Surprisingly, even small differences in TMB were independent of time between primary and metastatic tumor tissue (R^2^=1.025E-4, p=0.969; figure 2B) indicating that in general TMB does not accumulate significantly over time.

### Dissimilar lymphocyte infiltration between primary and metastatic melanoma lesions

**Figure 3:**
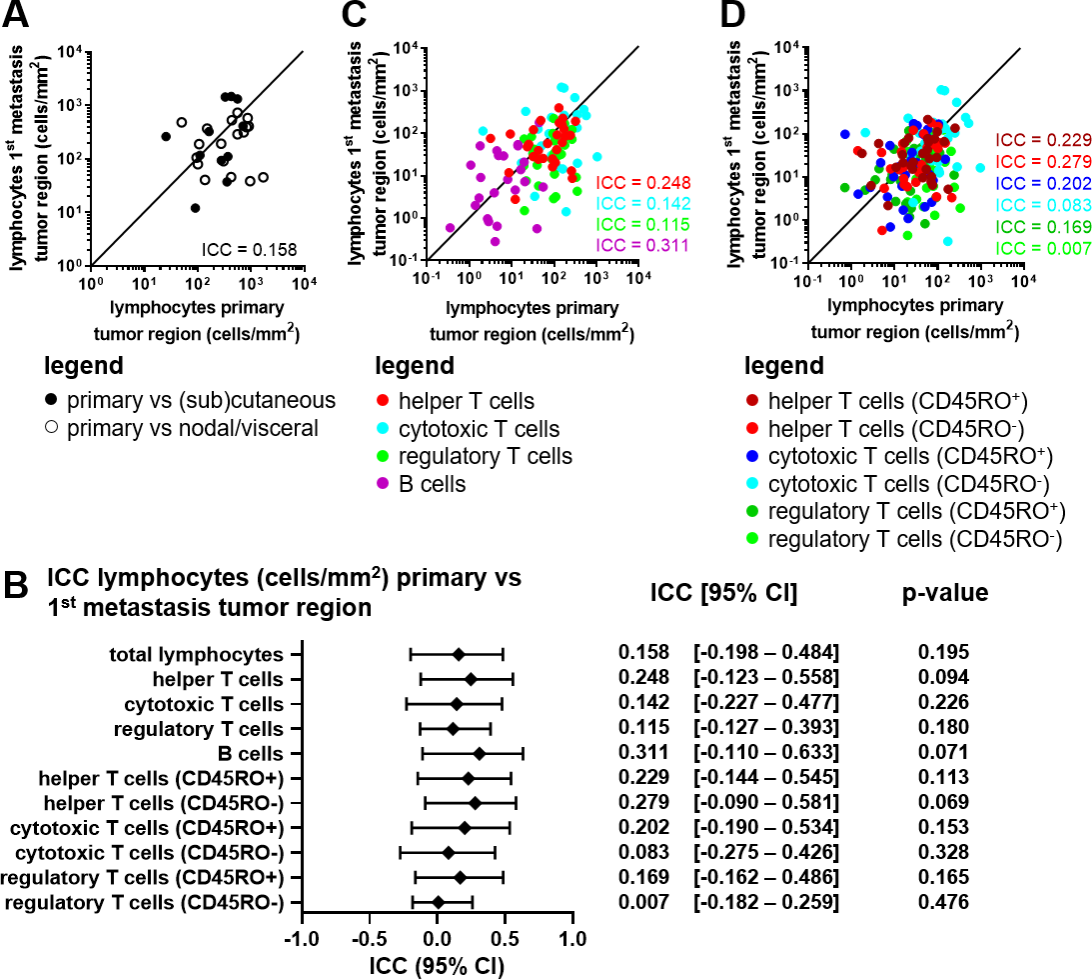
Analysis of lymphocyte subsets in paired primary and metastatic melanoma lesions. (A) Correlation plot of the total lymphocyte density (cells/mm^2^) in the tumor region of the primary tumor versus the first metastatic lesion. Closed dots represent paired primary cutaneous tumors with metastatic tumors at (sub)cutaneous locations. Open dots represent paired primary cutaneous tumors with metastatic tumors at nodal or visceral locations. (B) ICC value, 95% CI and p value per phenotype in the tumor region of the primary tumor versus the first metastatic lesion. (C) Correlation plot of the lymphocyte subset densities (cells/mm^2^) in the tumor region of the primary tumor versus the first metastatic lesion. (D) Correlation plot of the lymphocyte CD45RO^+/-^ subset densities (cells/mm^2^) in the tumor region of the primary tumor versus the first metastatic lesion. (A–D) Concordance is shown and quantified using the ICC. n=29. ICC, intraclass correlation coefficient.

Efficacy of ICI might also be dependent on the presence of TILs possibly recognizing mutation-derived neoantigens. Since the number of mutations (TMB) was similar between primary tumor and metastases, we hypothesized that, as a result of immunosurveillance, the number of TILs might also be similar. With mIHC, lymphocyte subsets were studied in ROI: tumor, invasive margin, and tumor +invasive margin. In contrast to the TMB, poor agreement was observed for the total lymphocyte density (cells/mm^2^) within the tumor of primary lesions compared with the first available metastatic lesions of individual patients (ICC=0.158; figure 3A–B). The same held for total lymphocyte density between invasive margin, or tumor + invasive margin (online supplemental figure 2A-C). Similarly, lymphocyte subset densities varied between the tumor of primary and metastatic lesions (ICC_helper T cells_ = 0.248, ICC_cytotoxic T cells_ = 0.142, ICC_regulatory T cells_ = 0.115, ICC_B cells_ = 0.311; figure 3B–C). Only a modest agreement between cytotoxic T cell densities in the invasive margin of the primary lesion versus the metastatic lesion was observed (ICC_cytotoxic T cells_ = 0.525; online supplemental figure 2C, D), however this was lost when taking tumor + invasive margin into account (ICC_cytotoxic T cells_ = 0.323; online supplemental figure 2C, E). Dichotomizing T lymphocyte subsets into CD45RO positive and negative subsets did not improve ICC values in the tumor (figure 3B and C) except for CD45RO^+^ cytotoxic T cells in the invasive margin (ICC_cytotoxic T cells (CD45RO+)_ = 0.568; online supplemental figure 2C, F) and tumor + invasive margin (ICC_cytotoxic T cells (CD45RO+)_ = 0.450; online supplemental figure 2C, G).

10.1136/jitc-2021-004329.supp4 Supplementary data

### Lymphocyte densities are similar between distinct metastatic melanoma lesions of individual patients

**Figure 4:**
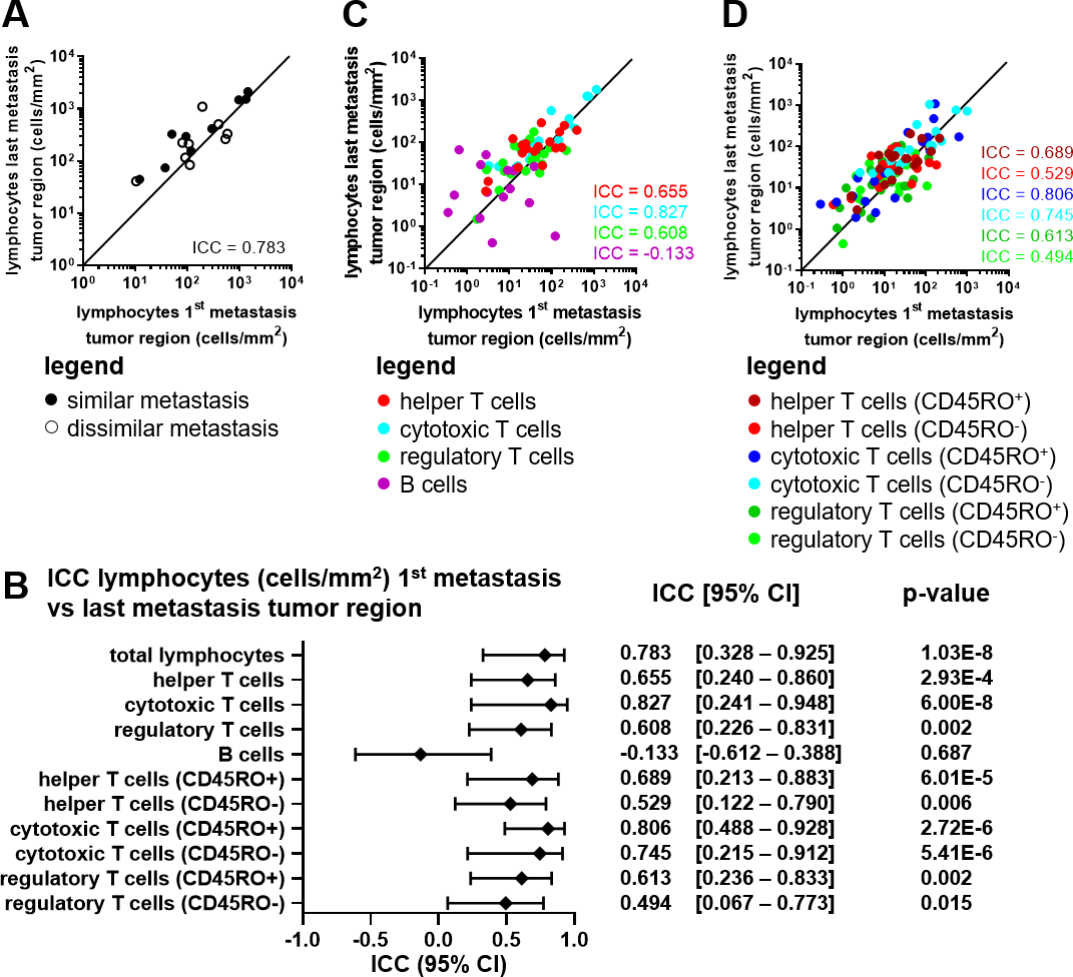
Lymphocyte subsets in paired in early and late metastatic melanoma samples. (A) Correlation plot of the total lymphocyte density (cells/mm^2^) in the tumor region of the first metastatic lesion versus the last metastatic lesion. Closed dots represent paired metastatic lesions similar anatomically locations. Open dots represent paired metastatic lesions at different anatomical locations. (B) ICC value, 95% CI and p value per phenotype in the tumor region of the first metastatic lesion versus the last metastatic lesion. (C) Correlation plot of the lymphocyte subset densities (cells/mm^2^) in the tumor region of the first metastatic lesion versus the last metastatic lesion. (D) Correlation plot of the lymphocyte CD45RO^+/-^ subset densities (cells/mm^2^) in the tumor region of the first metastatic lesion versus the last metastatic lesion. (A–D) Concordance is shown and quantified using the ICC. n=18. All metastatic lesions are acquired before receiving ipilimumab. ICC, intraclass correlation coefficient.

The similarity between multiple metastatic tumors of individual patients was investigated by determining the lymphocytic infiltration and composition of two metastatic lesions, taken at different time points before receiving ICI (first and last metastatic lesion we had available). Different metastatic lesions were very comparable in overall lymphocyte density within the tumor region (ICC=0.783; figure 4A–B), the invasive margin, and the tumor +invasive margin (online supplemental figure 3A–C). Cytotoxic T cell density was the most stable subset across metastatic sites, followed by helper T cell, and regulatory T cell subsets (ICC_cytotoxic T cells_ = 0.827, ICC_helper T cells_ = 0.655, ICC_regulatory T cells_ = 0.603; figure 4B–C and online supplemental figure 3C–E). B cell densities showed a poor agreement between the multiple metastatic lesions (ICC_B cells_ = –0.133), and only agreed modestly when evaluating invasive margin (ICC_B cells_ = 0.400) and tumor +invasive margin (ICC_B cells_ = 0.487) (online supplemental figure 3C–E). Dichotomizing T lymphocyte subsets into CD45RO positive and negative subsets slightly weakened observed agreements on overall T cells but still showed modest to very good agreements for cytotoxic T cells and helper T cells (figure 4B and C and online supplemental figure 3C, F–G). An increase in CD45RO^+^ T lymphocytes was observed over time between the primary tumor and multiple metastatic lesions (online supplemental figure 4). Total TILs nor any of the lymphocytic subsets correlated to TMB value in the different tissue regions (online supplemental figure 5). Four metastases harbored a mutation in PTEN or genes of the β-catenin pathway (online supplemental table 2), which is described to influence T cell infiltration negatively.^50 51^ However, metastatic lesions with mutations in these genes did not contain less lymphocytes compared with metastatic lesions that did not harbor mutations in PTEN or genes of the β-catenin pathway (data not shown). In conclusion, we observed a clear correlation between TIL phenotypes and their density within different metastatic lesions of the same patient. However, it does not seem that overall TMB value directly has any influence on the number of TILs. These findings suggest that the TME of different anatomical sites metastatic lesions does not have any effect on the lymphocyte infiltration indicating that, not the supporting tissue, but the tumor itself is the dominant factor.

10.1136/jitc-2021-004329.supp5 Supplementary data

10.1136/jitc-2021-004329.supp6 Supplementary data

10.1136/jitc-2021-004329.supp7 Supplementary data

### HLA-ABC status of metastatic lesions is correlated with cytotoxic T cell infiltration, and TMB

**Figure 5:**
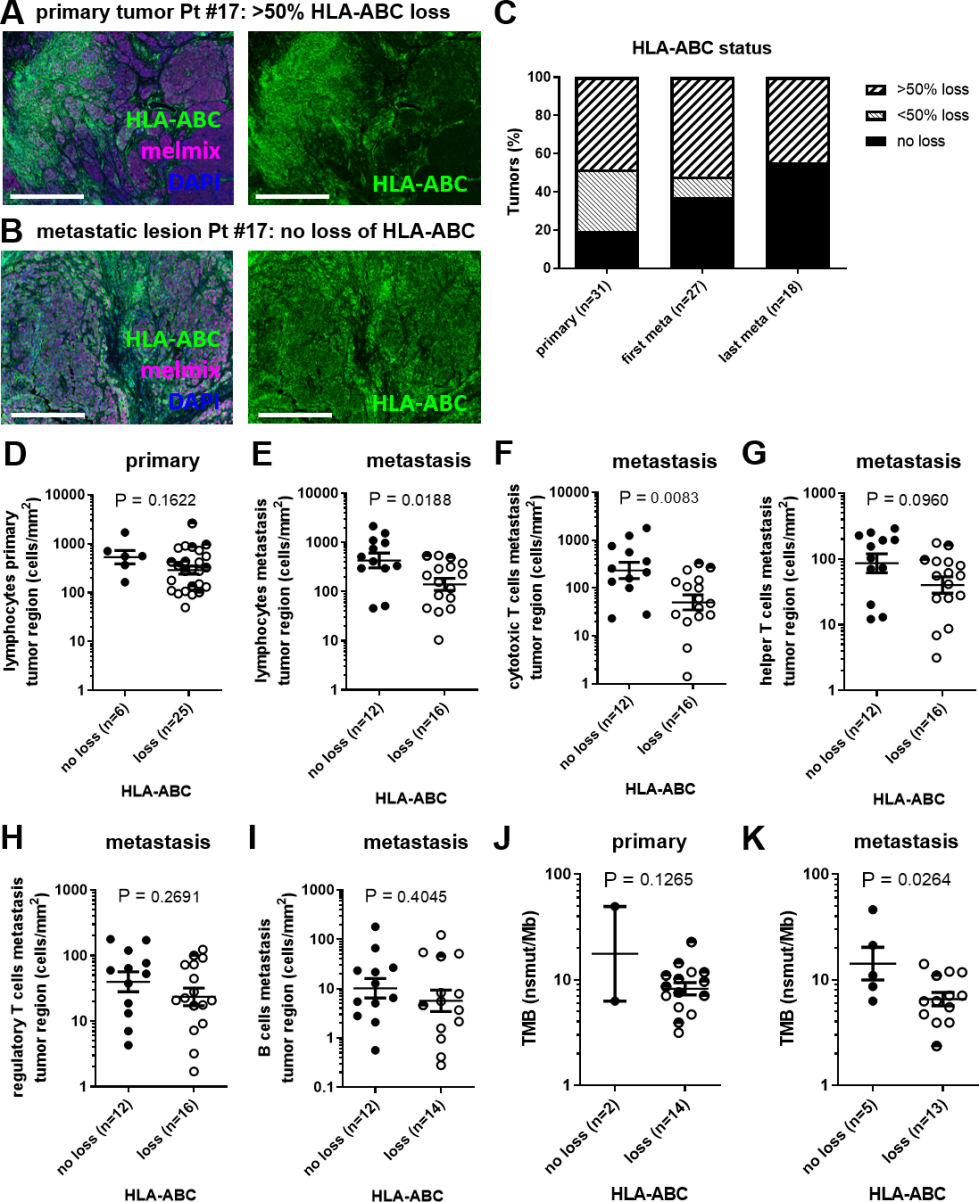
HLA-ABC status in relation to TIL and TMB. (A) Example of HLA-ABC expression (green) in the primary tumor of Pt #17 shown together with melmix (magenta) and DAPI (blue) (left) or only HLA-ABC (green) shown (right). Scale bars represent 0.5 mm. (B) Example of HLA-ABC expression (green) in a metastatic lesion of Pt #17 shown together with melmix (magenta) and DAPI (blue) (left) or only HLA-ABC (green) shown (right). Scale bars represent 0.5 mm. (C) HLA-ABC status of the primary tumor, first and last metastatic lesion. (D) HLA-ABC status in relation to overall lymphocyte infiltration in the primary tumor region. (E) HLA-ABC status in relation to overall lymphocyte infiltration in the metastatic tumor region. (F–I) HLA-ABC status in relation to infiltration of (F) cytotoxic T cells, (G) helper T cells, (H) regulatory T cells and (I) B cells in the metastatic tumor region, respectively. (J) HLA-ABC status in relation to TMB of the primary tumor. (K) HLA-ABC status in relation to TMB of the metastatic lesion. (D–K) Closed dots represent tumors without loss of HLA-ABC, open dots represent tumor with >50% loss of HLA-ABC and half-closed dots represent tumors with <50% loss of HLA-ABC. nsmut/Mb, non-synonymous mutations/mega base; Pt #, patient number; TIL, tumor infiltrating lymphocyte; TMB, tumor mutational burden.

MHC-I expression is important for the presentation of tumor-specific (neo)antigens to the cytotoxic T cells. HLA-ABC expression was therefore scored and analyzed in three different categories: no loss of HLA-ABC, less than 50% loss of HLA-ABC (<50%), and more than 50% loss of HLA-ABC (>50%) (figure 5A–B and online supplemental figure 6A, B). Primary tumors more often showed a degree of HLA-ABC loss than their metastatic counterparts (figure 5C). When only taking two categories into account, no loss of HLA-ABC and loss of HLA-ABC (either <50% or >50% loss of HLA-ABC), the HLA-ABC status of primary tumor versus the metastatic lesion showed a low agreement (corrected Cramér’s V=24.7%). The agreement of HLA-ABC status between the first and last metastatic lesions was substantially higher (corrected Cramér’s V=63.2%). No notable differences in HLA-ABC status between lymph node metastases and visceral metastases were observed (data not shown). In primary tumors, HLA-ABC status did not appear to correlate with TIL densities (figure 5D and online supplemental figure 6C, E). In metastatic lesions however, HLA-ABC loss correlated with lower TILs (no loss vs loss of HLA-ABC mean=426.580 vs 139.959 cells/mm^2^, p=0.0188; figure 5E and online supplemental figure 6D, F). Especially, the cytotoxic T cell population was decreased (no loss vs loss of HLA-ABC mean=233.884 vs 50.119 cells/mm^2^, p=0.0083; figure 5F and online supplemental figure 6G, K) while the other T lymphocyte subsets were not correlating to HLA-ABC status (figure 5G–I). B cells were significantly lower in the invasive margin when loss of HLA-ABC was observed (online supplemental figure 6J). We further analyzed whether TMB also correlated to HLA-ABC status (figure 5J–K). A more than twofold lower TMB was observed in metastatic lesions with a loss of HLA-ABC (no loss vs loss of HLA-ABC mean=14.22 vs 6.55 nsmut/Mb, p=0.0264; figure 5K). HLA-ABC status in the primary tumor did not correlate to TMB value (figure 5J), however we only had TMB data from two primary tumors that did not show a loss in HLA-ABC expression. In conclusion, metastatic lesions expressing HLA-ABC had higher TIL numbers, specifically high cytotoxic T cell numbers, and a higher TMB value.

10.1136/jitc-2021-004329.supp8 Supplementary data

### TMB and HLA-ABC status, but not TIL, are associated with clinical benefit or PFS on ipilimumab

**Figure 6:**
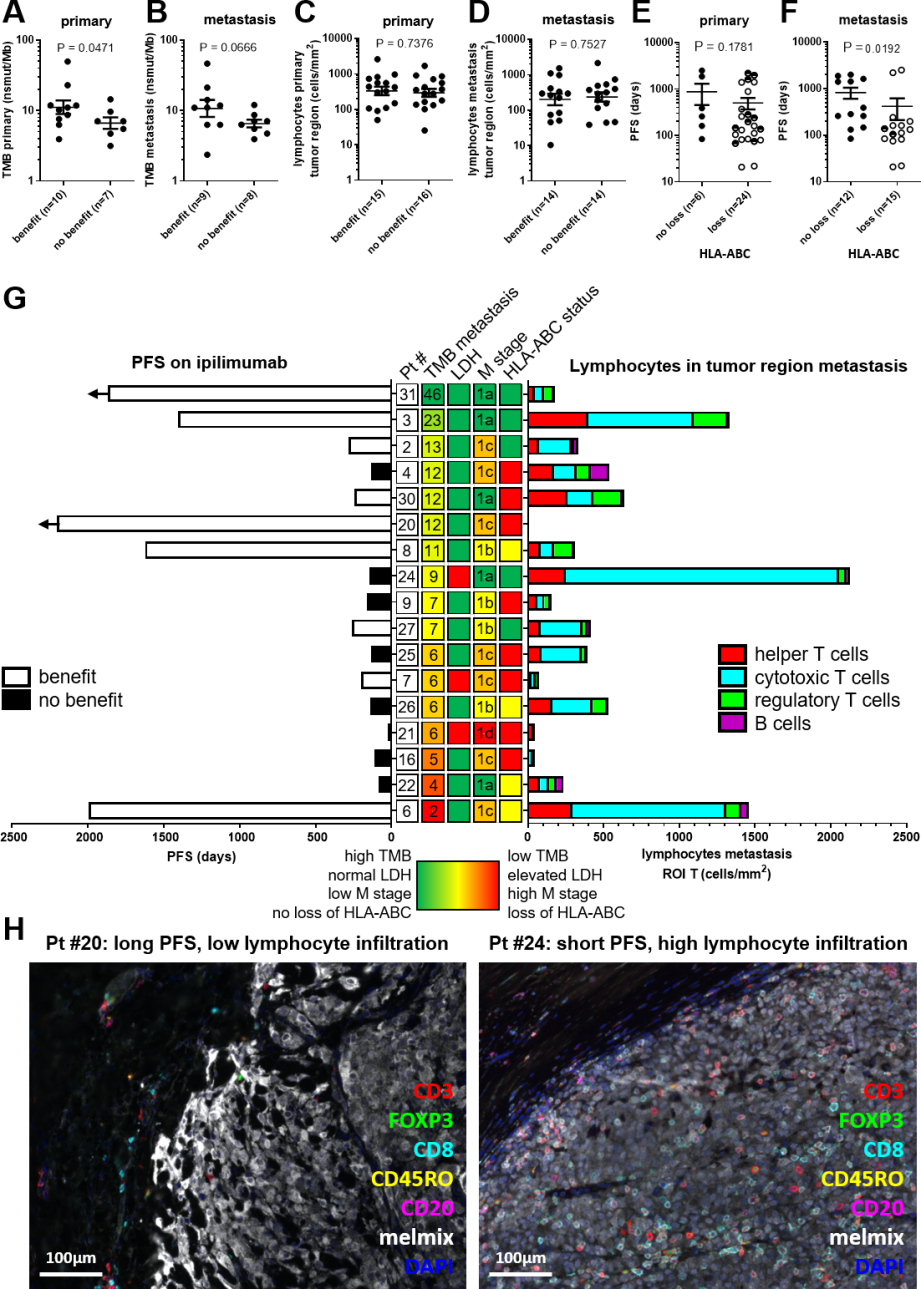
TMB, lymphocyte infiltration, HLA-ABC status, LDH status, and M stage in relation to progression-free survival on ipilimumab treatment. (A) TMB (nsmut/Mb) of the primary tumor for clinical benefit categories. (B) TMB of the metastasis for clinical benefit categories. (C) Overall lymphocyte infiltration in tumor region of the primary tumor in relation to clinical benefit. (D) Overall lymphocyte infiltration in tumor region of the last metastasis before ipilimumab in relation to clinical benefit. (E) HLA-ABC status of the primary tumor in relation to PFS on ipilimumab treatment. (F) HLA-ABC status of the metastatic lesion in relation to PFS on ipilimumab treatment. (E–F) Closed dots represent tumors without loss of HLA-ABC, open dots represent tumor with >50% loss of HLA-ABC and half-closed dots represent tumors with <50% loss of HLA-ABC. (G) Butterfly plot of 17 patients with melanoma with PFS on the left. Horizontal arrows indicate ongoing PFS. Lymphocyte infiltration of each patient in tumor region of a metastatic lesion is plotted on the right. Patients are ranked based on TMB (nsmut/Mb) of the metastatic lesion with high TMB (green) on top and low TMB (red) on the bottom. Pt # are on the left of TMB values in white boxes. On the right side of TMB value, LDH status, HLA-ABC status, and M stage at the start of ipilimumab treatment are shown in the middle. LDH green=LDH normal at start of treatment and LDH red=LDH elevated at start of treatment. M stage in green is M1a, yellow is M1b, orange is M1c, and red is M1d. HLA-status green=no loss of HLA-ABC, yellow=<50% loss of HLA-ABC and red=>50% loss of HLA-ABC. (H) example of a patient with a long PFS and low lymphocyte infiltration (left image) and an example of a patient with a short PFS but a high lymphocyte infiltration (right image). LDH, lactate dehydrogenase; M stage, metastatic stage; nsmut/Mb, non-synonymous mutations/mega base; PFS, progression-free survival; Pt #, patient number; ROI, regions of interest; TMB, tumor mutational burden.

To assess whether TMB, and/or TIL are associated with response to ipilimumab, patients were grouped according to the presence or absence of treatment benefit (defined as a complete response, partial response, or stable disease for at least 6 months). Clinical benefit of ipilimumab was associated with high TMB in primary tumors (benefit vs no benefit mean=11.12 vs 6.63 nsmut/Mb, p=0.0471; figure 6A) and in metastatic lesions (benefit vs no benefit mean=10.69 vs 6.54 nsmut/Mb, p=0.0666; figure 6B). The total number of TIL in the tumor or the invasive margin did not correlate with clinical benefit of ipilimumab (figure 6C–D). Similarly, the numbers of T helper cells, cytotoxic T cells, regulatory T cells, and B cells in the tumor or the invasive margin did not correlate with treatment outcome (online supplemental figure 7A, B). Next, we also investigated whether the HLA-ABC status of the primary or the metastatic lesion was associated with PFS on ipilimumab treatment (figure 6E–F). Loss of HLA-ABC in the metastatic lesions, but not loss in primary tumors, showed a significant correlation to a shorter PFS on ipilimumab (no loss vs loss of HLA-ABC mean=821.25 vs 415.87 days, p=0.0192; figure 6E–F). From a subset of patients, we had data available from TMB, TIL, and HLA-ABC status, which was visualized together with other prognostic values such as lactate dehydrogenase (LDH) and M stage before start of treatment (figure 6G). High TMB clearly related to longer PFS (figure 6E and online supplemental figure 7B, C). When evaluating individual patients, it is striking to see that TIL density can vary tremendously independent of TMB. We observed patients with a long PFS and considerable high TMB but very low TIL density and vice versa, patients with a short PFS and low TMB but high lymphocyte infiltration (figure 6G–H). This suggests that TMB and TIL are unrelated and of different relevance for the outcome of immunotherapy.

10.1136/jitc-2021-004329.supp9 Supplementary data

## Discussion

In this study, a high degree of similarity in TMB between paired primary melanomas and metastatic lesions was observed. In contrast, the degree of lymphocyte infiltration and HLA-ABC status is highly variable between primary tumors and metastases. Strikingly, similar numbers of lymphocytes were observed in paired metastatic lesions irrespective of the organs/sites of origin. This similarity across metastases of individual patients is strongest for cytotoxic T cells, followed by helper T cells, and regulatory T cells. B cell density, however, agreed poorly between paired metastatic lesions. Furthermore, HLA-ABC was also more similar in different metastatic lesions of the same patient compared with the primary tumor. Loss of HLA-ABC correlated with lower overall TILs, cytotoxic T cells, and lower TMB value in metastatic lesions. High TMB, in either the primary tumor or metastases, directly correlated with clinical benefit of ipilimumab treatment. Loss of HLA-ABC in metastatic lesions correlated to a shorter PFS. Surprisingly, no such correlations were observed for lymphocyte infiltration.

It is important to assess the consistency of biological features during disease progression in view of expanding immunotherapy treatment options to the (neo)adjuvant setting, as well as for patients with early-stage disease. TMB was highly similar in primary tumors and metastatic lesions, independent of the time interval. This similarity is conflicting with the idea of mutation accumulation over time, especially considering the time interval of, on average 6 years (range=0.2–17.7 years) between the primary tumor and the metastatic tumor in this cohort of patients. A previous study, investigating TMB in unpaired primary and metastatic samples of non-small cell lung cancer did find a higher TMB in the metastatic setting.^52^ Another study, exploring TMB data of 121 matched tumor samples of different origin (only four paired melanoma samples), identified similar TMB in metastatic and primary tumors, with slightly higher TMB in metastatic lesions.^53^ In line with our results, a recent meta-analysis of five other studies also reported no difference in TMB between primary tumors and metastatic lesions, but this meta-analysis did not include any melanoma samples.^54^ For the treatment of patients with melanoma in the adjuvant setting, the TMB can therefore be determined on archived primary samples.

TMB analysis, for selecting immunotherapy eligibility or outcome prediction, has been done on metastatic tissue or primary tumor tissue. In most clinical trials, only one tumor is assessed for TMB, the primary tumor, a metastatic lesion, or an unspecified lesion.^11–13 55 56^ In this cohort of patients with melanoma treated with ipilimumab, a higher TMB was associated with clinical benefit, regardless of being derived from the primary tumor or a metastatic tumor. Although for metastatic tumors there was a clear trend, the correlation was not significant which could be due to the small sample size. These results are in line with previous studies that correlated TMB with response to ipilimumab based on either primary tumors or unspecified melanoma samples.^11 12^ However, because patient with low TMB could still respond to ICI therapy as also exemplified in this study (figure 6G and online supplemental figure 7C, D), TMB by itself is still insufficient to include or exclude individual patients from receiving ICI.^16^

In theory, a high TMB increases the amount of (neo)antigens, leading to a highly immunogenic tumor. As a result, a higher number of TIL might be expected.^57^ Here we do not find such correlation between the TMB and lymphocyte infiltration, nor any lymphocyte subset in particular, in primary or metastatic melanoma lesions. Most studies investigated the link between TMB or neoantigen load and the presence of cytotoxic T cell-related transcripts. A correlation between TMB and cytolytic transcriptional activity was found for stomach cancer and lung adenocarcinoma, however, this was not significant for melanoma.^58^ Our findings are supported by another study that specifically focused on melanoma. These investigators also did not find a correlation between TMB and a T cell–inflamed gene signature.^59^ Yet, a recent study analyzing multiple cancer types did find a weak but significant positive correlation of r=0.3 between neoantigen load and presence of cytotoxic T cell-related transcripts in patients with melanoma.^16^ However, in most other tumor types analyzed this correlation was not found.^16^ Important differences with the aforementioned studies is that TIL density in the current study is measured in spatial relation to the tumor, enabling us to give a more precise estimate of the number of TILs present and their location. We believe these results underline the multifactorial nature of requirements necessary for an effective antitumor response, and that TIL density is not a direct result of TMB alone.

Lymphocyte infiltration is likely a very dynamic process, changing over time. In this cohort of patients, there was, on average, almost 6 years between the primary tumor and the first metastatic lesion, whereas for paired metastatic lesions this was a little over 1 year. This shorter time interval could explain why multiple metastasis of individual patients had comparable lymphocyte infiltrations whereas these differed between primary and metastatic lesions. To the best of our knowledge this is the first time that this type of detailed immune cell analysis is performed, directly comparing primary and different metastatic lesions to each other. Specific tissue regions could be analyzed, which was especially important for lymph node metastasis, only including the tumor and exclude surrounding normal lymph node tissue. This would have not been possible with traditional gene expression profiling techniques. A limitation of our current study is the lack of information on the myeloid compartment and other factors such as fibroblasts, vessels, and hypoxia, which could also be important factors in the response to ipilimumab treatment. Therefore, a similar analysis between primary and metastasis would be interesting, especially since most previous studies focus on TILs only. A recent meta-analysis of Zou *et al* included 12 studies that investigated TIL density by scoring H&E slides (none melanoma). TIL numbers were discordant between primary and metastatic lesions in 39% of the cases, more often an increase in TIL in metastasis was observed.^54^ Two other studies investigated immune cell phenotypes by mIHC and showed heterogeneous TIL infiltration patterns between primary and multiple metastatic lesions, but only included 1–2 patient cases.^60 61^ Another study did not directly compare the immune cell infiltration between the different lesions.^62^ Here, we recommend that for setting up prediction models it is of importance to note that lymphocyte density is not interchangeable between primary and metastatic lesions, while it is similar in different metastatic lesions when specific tumor regions are considered.

Antitumor cytotoxic T cell responses can be hampered by the downregulation of MHC-I, a molecule essential for presentation of tumor specific (neo)antigens.^63^ This downregulation of MHC-I, a well-known tumor escape mechanism, might occur during tumor progression. Many studies have investigated the expression of MHC-I, or different components of the MHC-I antigen presentation machinery in primary and metastatic melanoma.^63^ However, not much is known about MHC-I expression in patient-paired primary and metastatic lesions derived from different anatomical sites. An overall lower MHC-I expression in metastases than in primary melanomas was reported, although in 6 out of the 21 patients that were analyzed it was reversed.^64^ This heterogeneity in MHC-I expression was confirmed in a case report.^65^ Our results emphasize that HLA-ABC expression can be different between the primary tumor and different metastatic lesions. HLA-ABC expression positively correlating with TILs has been reported for several tumor types including melanoma.^66 67^ Although it has been observed in primary melanomas,^68 69^ our data only confirms this for metastatic lesions. Furthermore, the retention of HLA-ABC expression coinciding with favorable PFS observed here was previously found for ipilimumab specifically but not for nivolumab (an anti-PD-1 monoclonal antibody).^28^ A difficulty in comparing results from different studies results from different types of scoring systems used to determine HLA-ABC expression. Standardized analysis software to determine exact expression of HLA-ABC, could solve this problem in future studies.

No significant associations between lymphocyte (subset) infiltration and outcome of ipilimumab treatment were observed in any of the tissue regions studied. This contrasts with the association between TMB and clinical benefit but is in line with the lack of correlation between TMB and lymphocyte infiltration. High pretreatment TIL has previously been reported as beneficial to ipilimumab response.^70^ Contradicting results are reported regarding lymphocyte subsets as important predictors for response. In some studies CD8^+^ T cells and not FOXP3^+^ T cells correlated with clinical benefit from ipilimumab,^71 72^ while another study found the exact opposite.^35^ Yet another study reported a low density of CD8^+^ T cells in combination with high PD-L1^+^CD163^+^ macrophages in the invasive margin to be important.^73^ These apparently conflicting findings may partially be attributed to the various methods used to determine immune cell infiltration. Some studies use H&E slides to score TILs,^35 70^ while others perform multiple IHC on consecutive slides or mIHC for the identification of particular immune cell subsets.^35 70–73^ Besides the type of tumor samples used, like primary or metastatic lesions, the regions of the tumor examined to score TILs has a major impact on the results; the invasive margin can have a very different immune cell density compared with the core of a tumor.^74 75^ This should be taken into account, especially when tumor samples from various locations are analyzed, such as visceral, nodal tumor resections, and also very small biopsies (possibly without an analyzable invasive margin). In melanoma, only few studies distinguish between the tumor core and the invasive margin.^39 72 73^ B cells have also gained a lot of interested in cancer research as they have been associated with favorable prognosis.^76^ Recently, particular B cell formations designated as tertiary lymphoid structures have been associated with response on anti-PD-1 and to a lesser extent anti-CTLA-4 immunotherapy.^77^ However, in this study, the density of B cells did neither in the invasive margin nor in the tumor correlate with response to ipilimumab. In conclusion, these conflicting results demonstrate that lymphocyte infiltration itself is not a reliable parameter to be applied for clinical decision-making. Correlating immune cell infiltration and response to ICI treatment has so far mostly focused only on the mere presence of immune cells within the TME. However, the functional state of immune cells could be a more discriminating predictor of response to therapy. We believe that incorporating functional markers into multiplex assays, such as immune checkpoints, cytokine/chemokine (receptors), and other markers related to the (dys)functionality of immune cells, may provide more insight in the aptness of immune cells and could be a more accurate predictor of response to therapy.

In summary, we here demonstrate that TMB is stable across primary and metastatic lesions in patients with melanoma. This supports the use of TMB derived from either primary or metastatic lesions as a valuable candidate contributing factor in prediction models for outcome of immunotherapy in patients with melanoma. HLA-ABC status in the metastatic lesion could to some extent contribute to a prediction model, but needs to be validated in independent cohorts. By contrast, lymphocyte infiltration differs substantially between the primary tumor and metastatic lesions, hampering its suitability for biomarker development, especially for patients with early-stage disease of whom no metastatic material is readily available. The stability of the TMB, in contrast to the variability of lymphocyte infiltration over time, between primary tumors and metastases, underlines the complexity of the TME. To develop clinical meaningful biomarkers, it is crucial future research takes this complexity into account. Finally, our finding that both lymphocyte infiltration and HLA expression are highly similar in different metastases, even at different anatomical locations, strongly indicates that the tumor itself is the driving factor and not the supporting tissue in the TME or the organ specific cells. We believe that this is an important finding and this also validates that biopsies can be taken from any anatomical site to get reliable data on TMB, HLA-ABC expression, and lymphocyte infiltration.

10.1136/jitc-2021-004329.supp10 Supplementary data

## Data Availability

Data are available upon reasonable request.
