# Supplementary figures and images for: Paired primary and metastatic lesions of patients with ipilimumab-treated melanoma: high variation in lymphocyte infiltration and HLA-ABC expression whereas tumor mutational load is similar and correlates with clinical outcome
Source: J Immunother Cancer. 2022 May 11;10(5):e004329. doi: 10.1136/jitc-2021-004329 (PMC9109111; doi:10.1136/jitc-2021-004329)

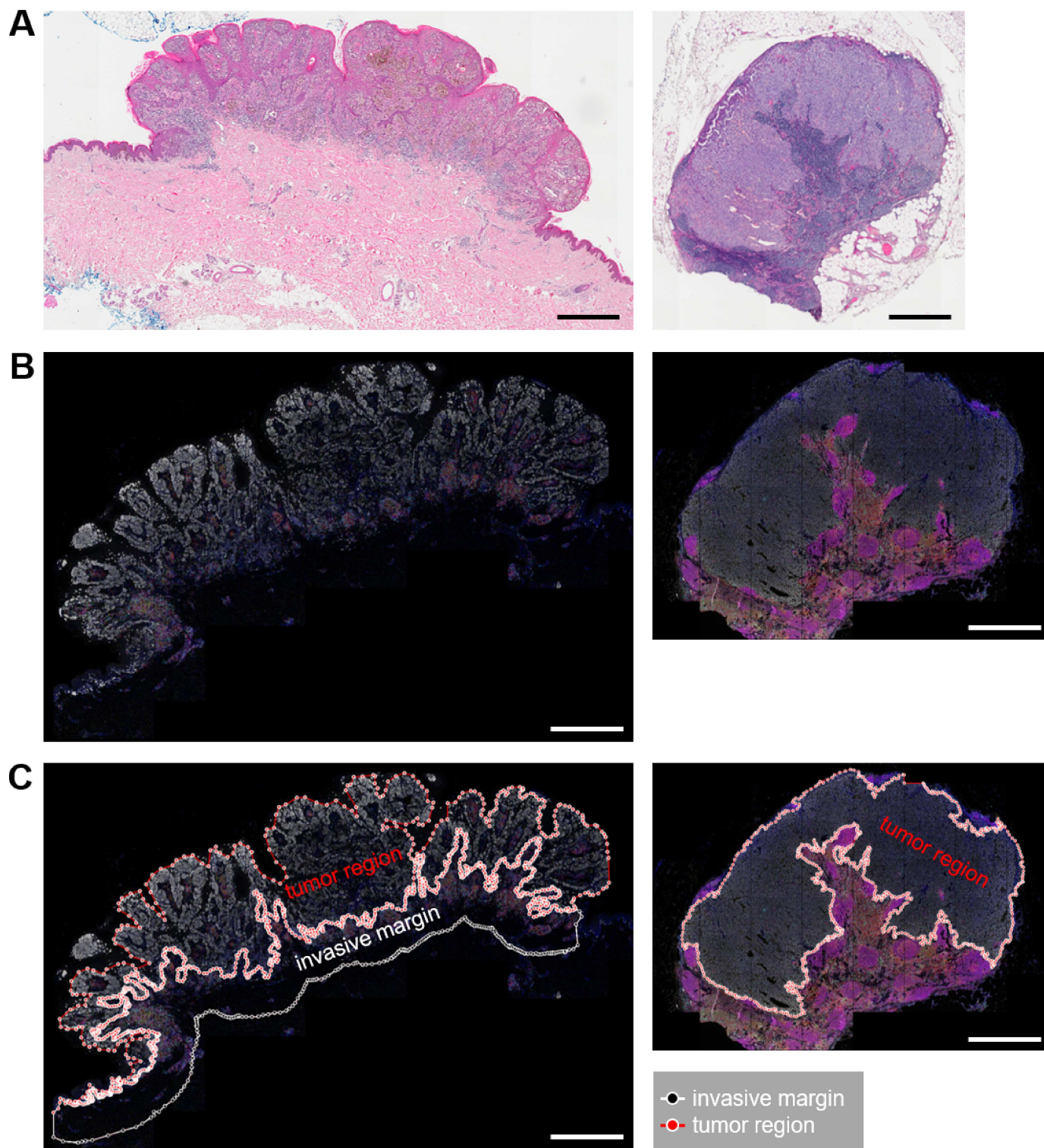

Supplement: Supplementary data [file jitc-2021-004329supp003.pdf]

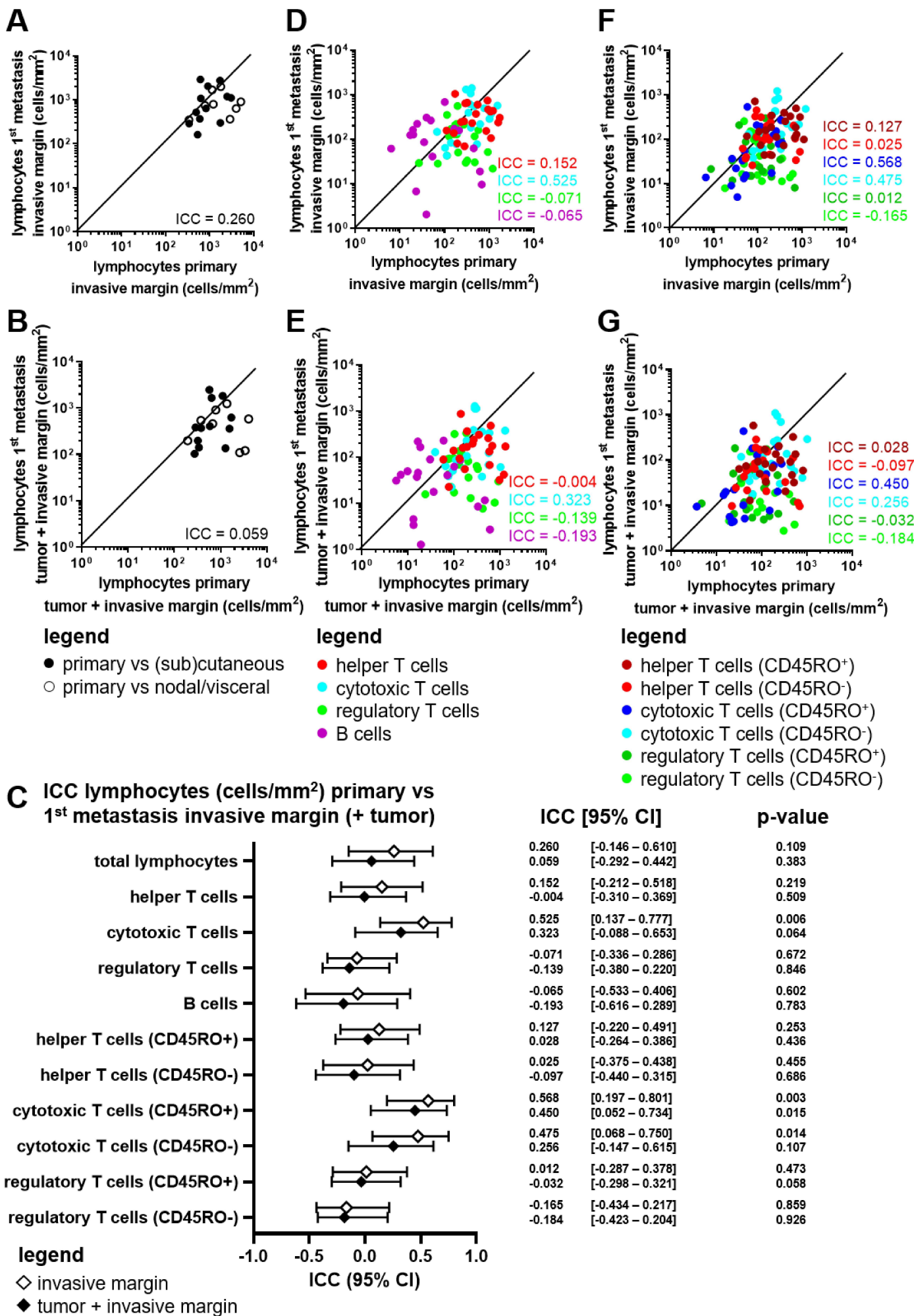

Supplement: Supplementary data [file jitc-2021-004329supp004.pdf]

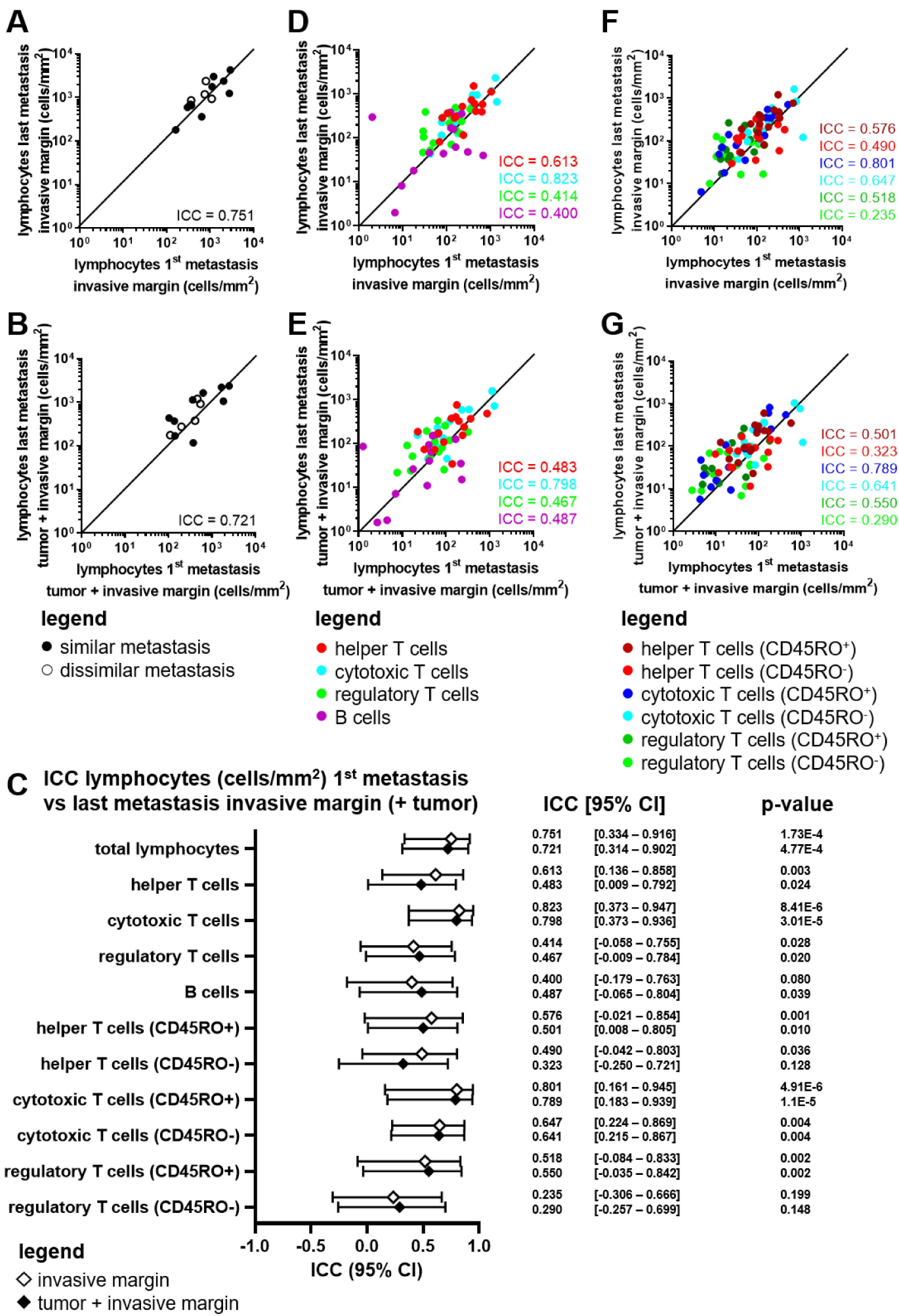

Supplement: Supplementary data [file jitc-2021-004329supp005.pdf]

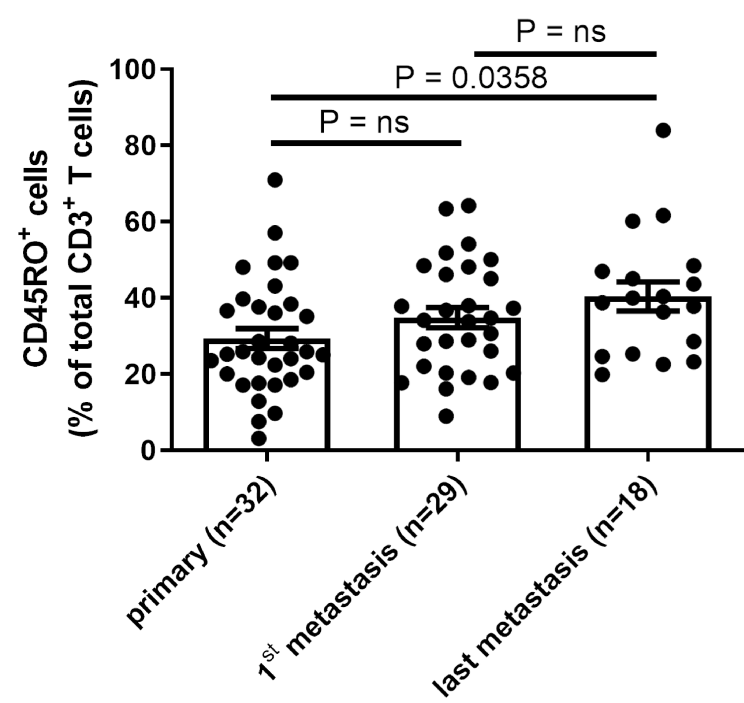

Supplement: Supplementary data [file jitc-2021-004329supp006.pdf]

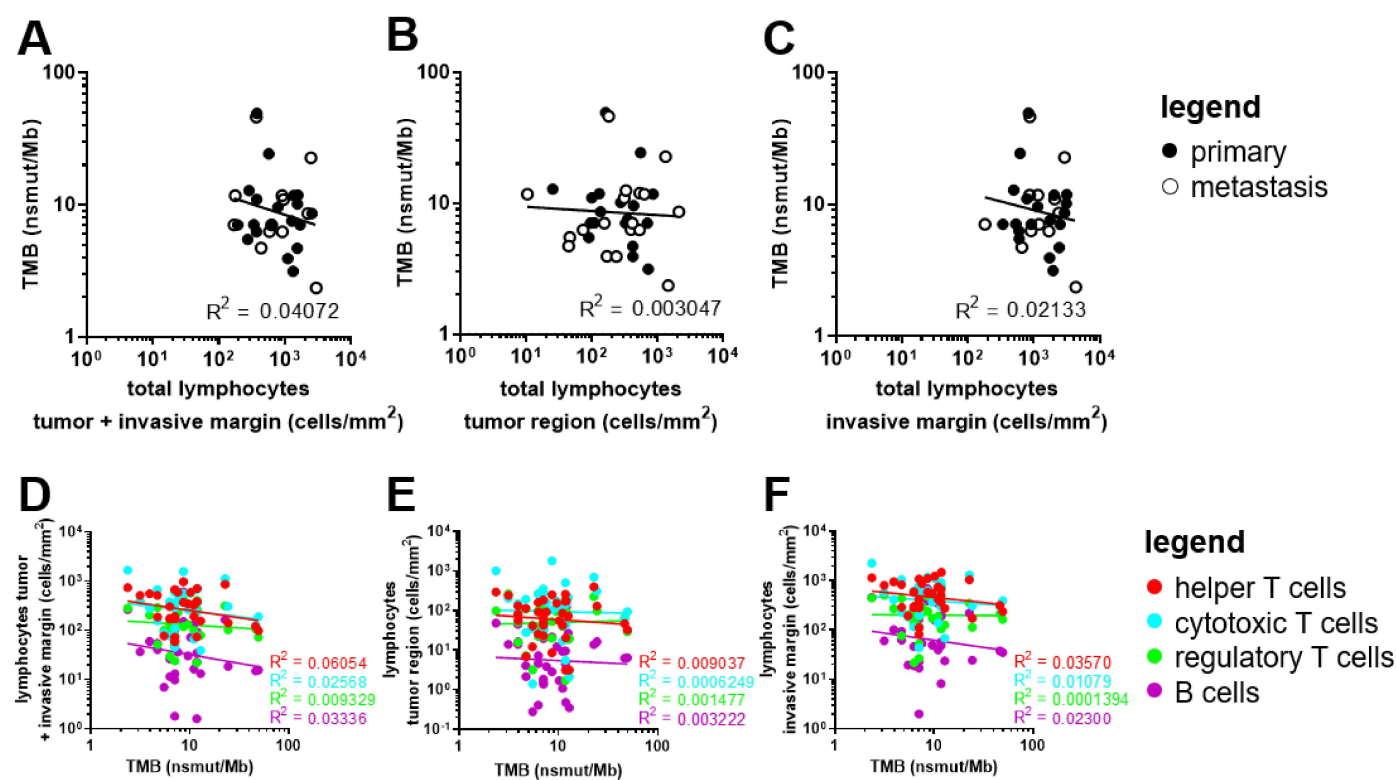

Supplement: Supplementary data [file jitc-2021-004329supp007.pdf]

**A** primary tumor Pt #14: >50% HLA-ABC loss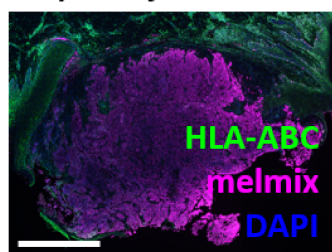**B** metastatic lesion Pt #14: >50% HLA-ABC loss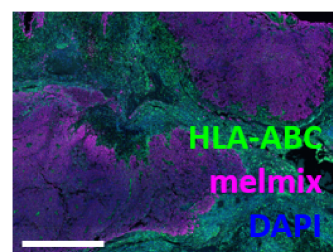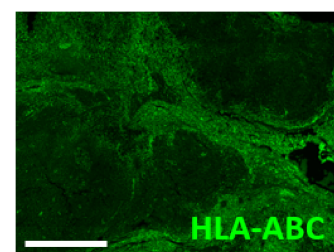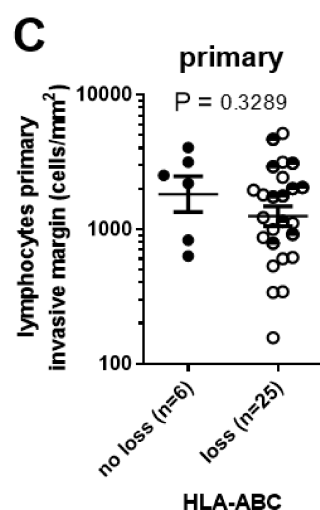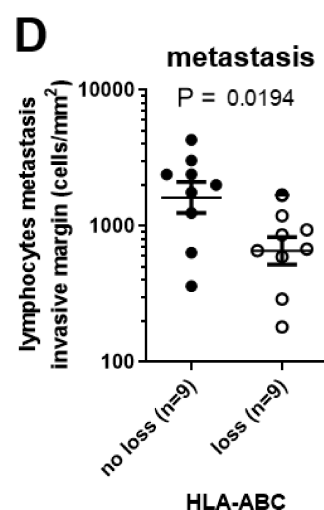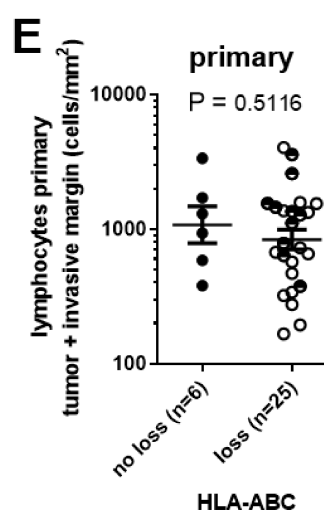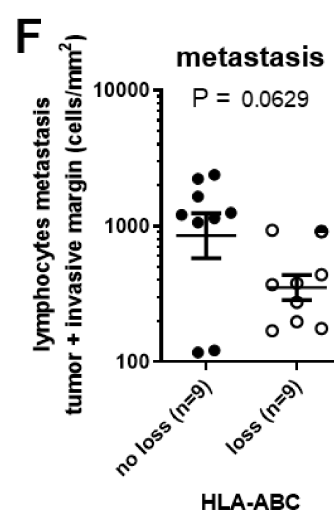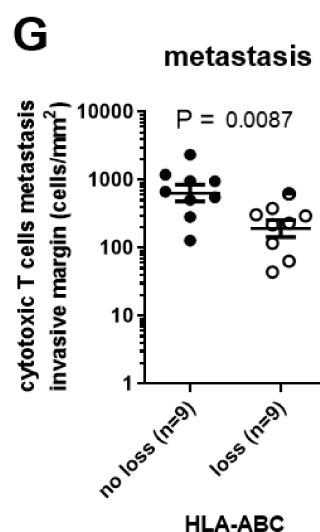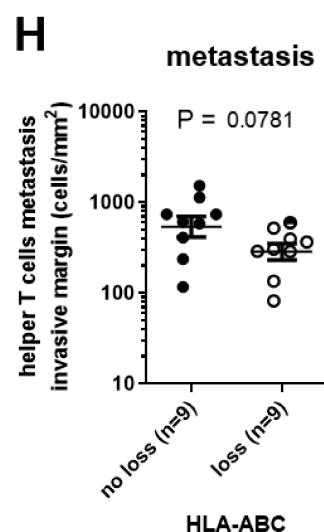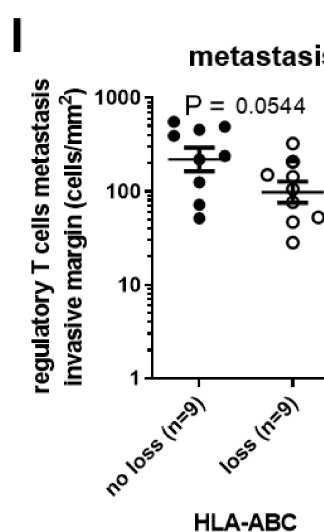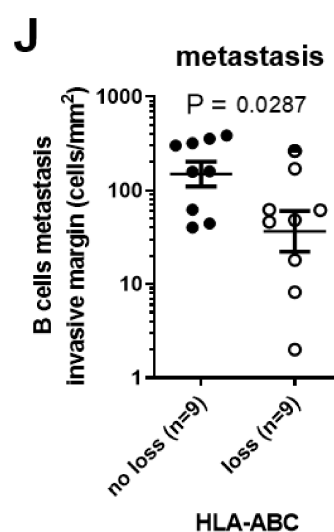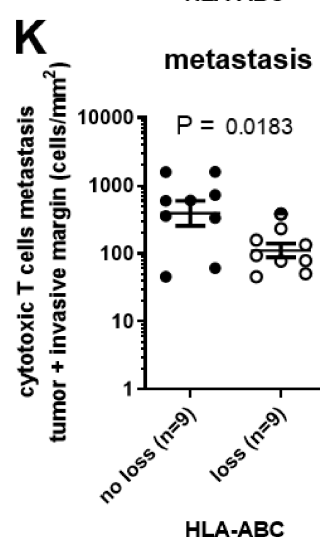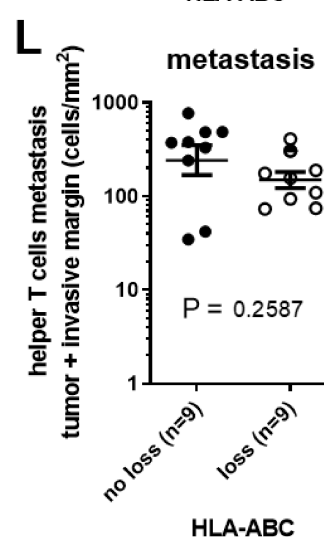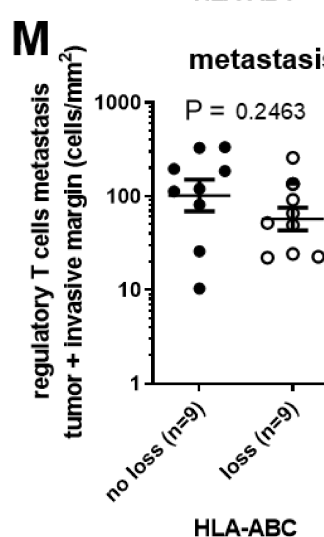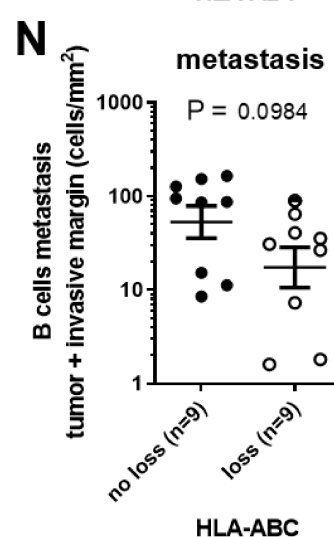

Supplement: Supplementary data [file jitc-2021-004329supp008.pdf]

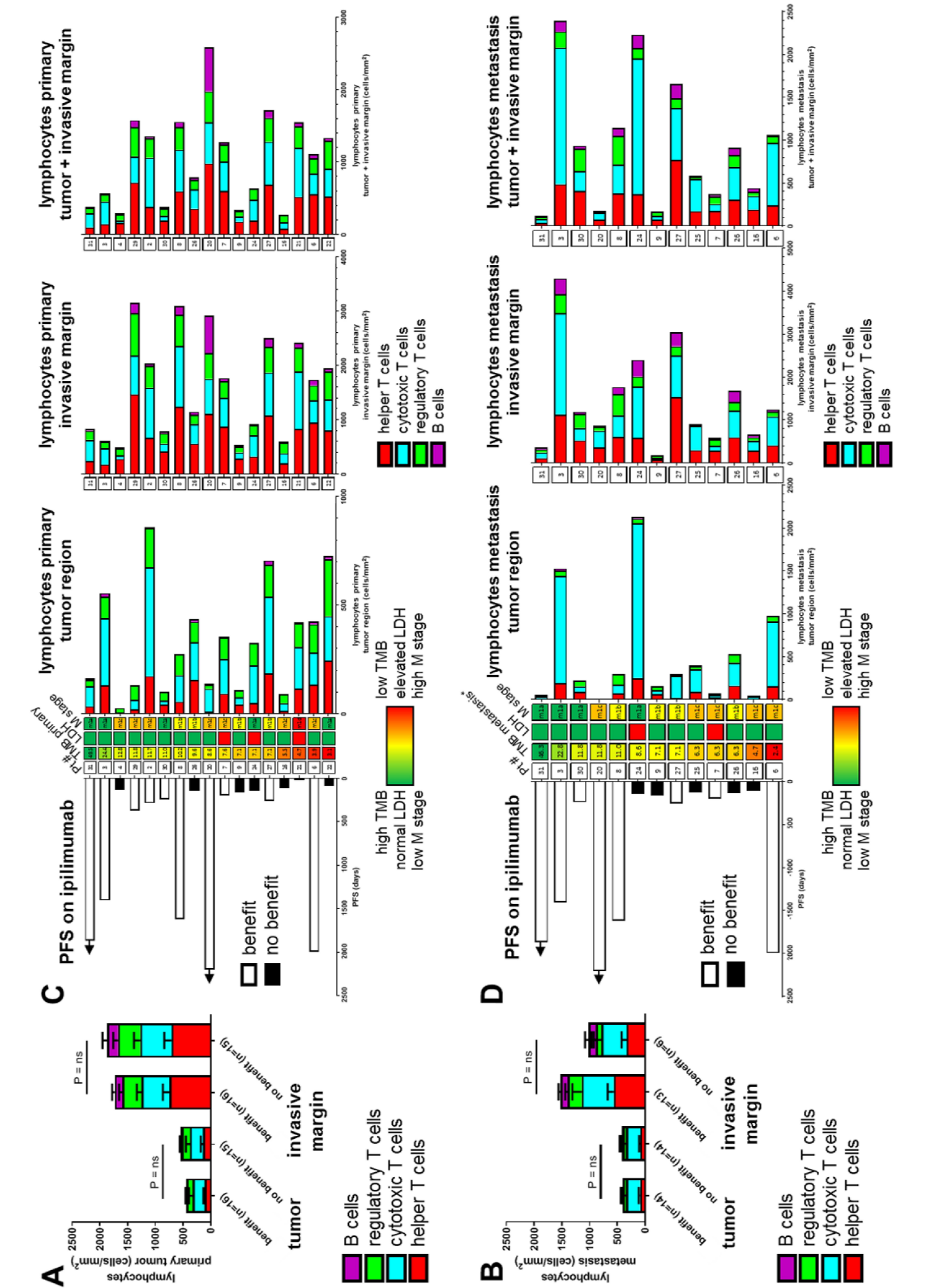

Supplement: Supplementary data [file jitc-2021-004329supp009.pdf]
