# Supplementary material for: Paired primary and metastatic lesions of patients with ipilimumab-treated melanoma: high variation in lymphocyte infiltration and HLA-ABC expression whereas tumor mutational load is similar and correlates with clinical outcome
Source: J Immunother Cancer. 2022 May 11;10(5):e004329. doi: 10.1136/jitc-2021-004329 (PMC9109111; doi:10.1136/jitc-2021-004329)
Supplement: Supplementary data [file jitc-2021-004329supp010.pdf]

Supplemental figure 1: Selection of tumor and invasive margin. (A) HE slide of a primary melanoma sample (left) and its respective metastatic melanoma lesion in the lymph node (right). (B) Spectral unmixed overview 20x images of primary tumor (left) and metastatic melanoma (right). (C) ROI for tumor and, if possible, invasive margin of ~0.5mm thickness, were manually selected on scanned tissues. Scale bars represent 1mm. HE, hematoxylin and eosin; ROI, region of interest.

Supplemental figure 2: Analysis of lymphocyte subsets in paired primary and metastatic melanoma lesions. (A) Correlation plot of the total lymphocyte density (cells/mm2) in the invasive margin of the primary tumor versus the first metastasis lesion. (B) Correlation plot of the lymphocyte subset densities (cells/mm2) in the tumor + invasive margin the primary tumor versus the first metastasis lesion. (A-B) Closed dots represent paired primary cutaneous tumors with metastatic tumors at (sub)cutaneous locations. Open dots represent paired primary cutaneous tumors with metastatic tumors at nodal or visceral locations. (C) ICC value, 95% CI and p-value per phenotype in the invasive margin (open diamonds) or the tumor + invasive margin (closed diamonds) of the primary tumor versus the first metastatic lesion. (D) Correlation plot of the lymphocyte subset densities (cells/mm2) in the invasive margin the primary tumor versus the first metastasis lesion. (E) Correlation plot of the lymphocyte subset densities (cells/mm2) in the tumor + invasive margin of the primary tumor versus the first metastasis lesion. (F) Correlation plot of the lymphocyte CD45RO+/- subset densities (cells/mm2) in the invasive margin of the primary tumor versus the first metastasis lesion. (G) Correlation plot of the lymphocyte CD45RO+/- subset densities (cells/mm2) in the tumor + invasive margin of the primary tumor versus the first metastasis lesion. (A-G) Concordance is shown and quantified using the ICC. n=20. CI, confidence interval; ICC, intraclass correlation coefficient.

Supplemental figure 3: Lymphocyte subsets in paired metastatic melanoma samples. (A) Correlation plot of the total lymphocyte density (cells/mm2) in the invasive margin of the first metastatic lesion versus the last metastatic lesion. (B) Correlation plot of the lymphocyte subset densities (cells/mm2) in the tumor + invasive margin of the first metastatic lesion versus the last metastatic lesion. (A-B) Closed dots represent paired metastatic lesions similar anatomically locations. Open dots represent paired metastatic lesions at different anatomical locations. (C) ICC value, 95% CI and p-value per phenotype in the invasive margin (open diamonds) or the tumor + invasive margin (closed diamonds) of the first metastatic lesion versus the last metastatic lesion. (D) Correlation plot of the lymphocyte subset densities (cells/mm2) in the invasive margin of the first metastatic lesion versus the last metastatic lesion. (E) Correlation plot of the lymphocyte subset densities (cells/mm2) in the tumor + invasive margin of the first metastatic lesion versus the last metastatic lesion. (F) Correlation plot of the lymphocyte CD45RO+/- subset densities (cells/mm2) in the invasive margin of the first metastatic lesion versus the last metastatic lesion. (G) Correlation plot of the lymphocyte CD45RO+/- subset densities (cells/mm2) in the tumor + invasive margin of the first metastatic lesion versus the last metastatic lesion. (A-G) Concordance is shown and quantified using the ICC. n=14. All metastatic lesions are acquired before receiving ipilimumab. CI, confidence interval; ICC, intraclass correlation coefficient.

Supplemental figure 4: CD45RO status of T lymphocytes in paired primary and metastatic tumor tissues. Total CD3+ T cells were gated for CD45RO expression and compared in primary versus first metastasis versus last metastasis.

Supplemental figure 5: Lymphocyte infiltration versus TMB. (A) Correlation plot of total lymphocyte densities (cells/mm2) in the tumor + invasive margin versus TMB (nsmut/Mb). n=31. (B) Correlation plot of total lymphocyte densities (cells/mm2) in the tumor region versus TMB (nsmut/Mb). n=36. (C) Correlation plot of total lymphocyte densities (cells/mm2) in the invasive margin versus TMB (nsmut/Mb). n=31. Closed dots represent primary melanoma and open dots represent metastatic melanoma lesions. (D) Correlation plot of TMB (nsmut/Mb) versus lymphocyte subsets (cells/mm2) in the tumor + invasive margin. n=31. (E) Correlation plot of TMB (nsmut/Mb) versus lymphocyte subsets (cells/mm2) in the tumor region. n=36. (F) Correlation plot of TMB (nsmut/Mb) versus lymphocyte subsets (cells/mm2) in the invasive margin. n=31. nsmut/Mb, nonsynonymous mutations/mega base; TMB, tumor mutational burden.

Supplemental figure 6: HLA-ABC status in relation to TIL infiltration and TMB. (A) Example of HLA-ABC expression (green) in the primary tumor of Pt #14 shown together with melmix (magenta) and DAPI (blue) (left) or only HLA-ABC (green) shown (right). Scale bars represent 1mm. (B) Example of HLA-ABC expression (green) in a metastatic lesion of Pt #14 shown together with melmix (magenta) and DAPI (blue) (left) or only HLA-ABC (green) shown (right). Scale bars represent 1mm. (C) HLA-ABC status in relation to overall lymphocyte infiltration in the invasive margin of the primary tumor. (D) HLA-ABC status in relation to overall lymphocyte infiltration in the invasive margin the metastatic lesion. (E) HLA-ABC status in relation to overall lymphocyte infiltration in the invasive margin + tumor region of the primary tumor. (F) HLA-ABC status in relation to overall lymphocyte infiltration in the invasive margin + tumor region of the metastatic lesion. (G-J) HLA-ABC status in relation to infiltration of (G) cytotoxic T cells, (H) helper T cells, (I) regulatory T cells and (J) B cells in the invasive margin of the metastatic lesion respectively. (K-N) HLA-ABC status in relation to infiltration of (K) cytotoxic T cells, (L) helper T cells, (M) regulatory T cells and (N) B cells in the invasive margin + tumor region of the metastatic lesion respectively. (C-N) Closed dots represent tumors without loss of HLA-ABC, open dots represent tumor with >50% loss of HLA-ABC and half-closed dots represent tumors with <50% loss of HLA-ABC. Pt #, patient number.

Supplemental figure 7: Lymphocyte subset infiltration versus patient outcome. (A) Lymphocyte subset infiltration (cells/mm2) in tumor region and invasive margin of the primary tumor for clinical benefit categories. (B) Lymphocyte subset infiltration (cells/mm2) in tumor region and invasive margin of metastatic lesions of which invasive margins were present for clinical benefit categories. (C) Butterfly plot of 17 melanoma patients of primary tumor lymphocyte infiltration characterization. (D) Butterfly plot of 13 melanoma patients of which a metastatic lesion with invasive margin lymphocyte infiltration characterization was possible. (C-D) Lymphocyte infiltration of each patient in the tumor region, invasive margin, and tumor + invasive margin of the primary or metastatic lesion is displayed on the right. Patients are ranked based on TMB (nsmut/Mb) of the primary tumor or a metastatic lesion from that patient with high TMB (green) on top and low TMB (red) on the bottom. PFS is displayed on the left and horizontal arrows indicate ongoing PFS. Pt # are on the left of TMB values in white boxes. On the right side of TMB value, LDH status and M stage at the start of ipilimumab treatment are shown in the middle. LDH green = LDH normal at start of treatment and LDH red = LDH elevated at start of treatment. M stage in green is M1a, yellow is M1b, orange is M1c, and red is M1d. LDH, lactate dehydrogenase; M stage, metastatic stage;

nsmut/Mb, nonsynonymous mutations/mega base; Pt #, patient number; TMB, tumor mutational burden.
